# Vascular Endothelial Growth Factor Receptor Expression in Orbital Cavernous Malformations and Lymphatic Malformations

**Published:** 2021-03-31

**Authors:** Ann Q. Tran, Marissa K. Shoji, Alexandra Levitt, Wendy W. Lee

**Affiliations:** 1 Department of Ophthalmology, Bascom Palmer Eye Institute, University of Miami, Miami, FL; 2 Department of Ophthalmology, Manhattan Eye and Ear Throat Hospital, Northwell Health, New York, NY

**Keywords:** Orbital cavernous malformations, lymphatic venous malformations, vascular malformations, vascular lesions, vascular endothelial growth factor

## Abstract

**Purpose:**

To investigate the presence of vascular endothelial growth factor receptors (VEGFR) in orbital cavernous malformations and lymphatic malformations to further understand the feasibility of anti-VEGF treatment.

**Methods:**

This study was a single-center retrospective chart review performed at the Bascom Palmer Eye Institute of patients who underwent surgical excision of orbital cavernous malformations and lymphangiomas from 2000 – 2017. Immunohistochemical staining of these lesions for VEGFR1 and VEGFR2 expression was performed.

**Results:**

A total of 25 patients were identified with cavernous malformations (n=15) and lymphatic malformations (n=10). Ten specimens (7 cavernous malformations, 3 lymphatic malformations) underwent further immunohistochemical analysis. Six of 7 cavernous malformations and one of 3 lymphatic malformations stained positive for VEGFR1 and VEGFR2.

**Conclusions:**

Both cavernous malformations and lymphatic malformations appear to express VEGFR with varying frequency. Additional studies are needed to better characterize the pathogenesis of these lesions, nature of VEGFR expression, and potential efficacy of anti-VEGF treatment.

## Introduction

Cavernous and lymphatic malformations comprise a relatively high percentage of orbital lesions (Shields et al. 2004). Patients with these types of vascular malformations may clinically present with pain, edema, proptosis, restriction of eye movement, optic nerve compression, and amblyopia (Rootman et al. 2014 & Shoji et al. 2020). Although these lesions are not malignant, they have potentially serious sequelae, including mass effect on the globe and optic nerve, infection, and bleeding (Rootman et al. 2014, Elluru et al. 2014, Wiegand S et al. 2013, Woo et al. 2017).

Treatment of orbital vascular malformations can be challenging due to their location and risk of recurrence. In general, management is individualized based on the lesion type and location. Current treatment modalities include excisional or debulking surgery, drainage, sclerotherapy, and systemic therapy such as sirolimus (Shoji et al. 2020, Harris 1999, Wiegand et al. 2011, Macintosh et al. 2014, Cheng et al. 2015). Understanding the underlying pathophysiology may be beneficial to development of alternative treatment modalities.

Vascular endothelial growth factors (VEGF) are critical regulators of vascular and lymphatic function. They may be involved in the pathogenesis of these lesions and potentially offer a theoretical therapeutic target. Previous studies have examined the presence of VEGF receptors (VEGFR) in orbital tumors; however, these studies are small, and their results varied (Rootman et al. 2014, Nagasaka et al. 2007, Atchison et al. 2016). Therefore, this study aims to further characterize VEGFR expression in orbital cavernous and lymphatic malformations.

## Methods:

A retrospective, single-center study was conducted at the Bascom Palmer Eye Institute. The research protocol was approved by the Institutional Review Board, adhered to the tenets set forth by the Declaration of Helsinki, and was Health Insurance Portability and Accountability Act compliant. A total of 25 patients who underwent complete surgical excision of orbital cavernous malformations and debulking of lymphatic malformations from 2000 to 2017 were included. Demographic, lesion, surgical, and pathologic specimen data were collected. An ocular pathologist reviewed all images.

Ten cases underwent immunohistochemical analysis. Surgical specimens were sent to the Florida Lions Ocular Pathology Laboratory. Formalin-fixed paraffin-embedded specimens were prepared. Immunohistochemistry was performed with antibodies for VEGF Receptor 1 (1:200; Abcam Cat# ab32152, RRID: AB_778798) and VEGF Receptor 2 (1:200; Cell Signaling Technology Cat# 9698, RRID: AB_11178792). Heat-mediated antigen retrieval with fresh sodium citrate buffer was performed followed by standard immunohistochemistry protocol. Images were taken with the Evos FL Auto Imaging System (Thermo Fisher Scientific, Waltham, MA, USA).

## Results:

A total of 25 different patients were included in this study. 15 patients had cavernous malformations, (mean age 49.6±11.7 years old, 53% male). Patients presented with proptosis (66%), pain (26%), diplopia (13%), and compressive optic neuropathy (33%). Most lesions were intraconal (73%) and ovoid in shape with a uniform presentation. There was one case of bony erosion and once case of the lesion embedded within the temporalis muscle. All patients underwent complete surgical excision. The most common surgical approach based on the lesion location included a lateral orbitotomy approached from the lid crease (47%) lateral orbitotomy with bone flap (13%), swinging eyelid approach (20%), transcaruncular approach (13%) or subciliary approach (7%). Removal with cryotherapy was used in one case. No recurrences were noted after surgical excision. 7 specimens underwent immunohistochemical staining for VEGFR1 (6/7 positive) and VEGFR2 (6/7 positive, Table 1, Figure 1A–C).

A total of 10 patients with lymphatic malformations were identified (mean age 26.1±24.8 years old, 30% male). The lesions were located intraconal (40%) and involved the eyelid (50%). Two patients had prior hemorrhagic episodes. The patients presented with proptosis (60%), diplopia (40%), and pain (20%). One patient had symptoms of compressive optic neuropathy. One patient had enlargement with Valsalva maneuver. All patients underwent orbitotomy with debulking. Five patients had additional recurrences of symptoms after initial debulking prompting additional surgery (30%), injection of a sclerosing agent with interventional radiology (20%), trial of sildenafil (10%) and trial of steroid injection (10%). A total of 3 specimens underwent immunohistochemical staining for VEGFR1 (1/3 positive) and VEGFR2 (1/3 positive, Table 1, Figure (D – E):

## Discussion:

Due to their involvement in vascular and lymphatic development and function, VEGF and VEGFRs may play a key role in the pathogenesis and progression of orbital cavernous and lymphatic malformations. In our study, the majority of cavernous malformations demonstrated positive staining for VEGFR1/VEGFR2. However, our lymphatic malformations had lower rates of VEGFR1 and 2 expressions.

There are five structurally related mammalian VEGF ligands (VEGFA, VEGFB, VEGFC, VEGFD, and placenta growth factor PlGF), each of which has variants based on alternative splicing or processing (Tugues et al. 2011). There are three major VEGF receptors: VEGFR1, VEGFR2, and VEGFR3. While not evaluated in our study, VEGFR3 can bind to VEGFC and VEGFD and is involved in lymphatic endothelial cell function and lymphatic development (Tugues et al. 2011).

In our study, the majority of cavernous malformations demonstrated positive staining for VEGFR2. The VEGF pathway may have a pathophysiologic role in development and growth of cavernous malformations, and thus may be a target for therapy. VEGFR2 primarily contributes to angiogenesis through its interactions with VEGFA. VEGFR2 binds to VEGFA, VEGFB, and PlGF. The interaction between VEGFR2 and VEGFA is thought to be a key driver in endothelial cell proliferation and differentiation as well as vascular permeability (Tugues et al. 2011).

Additionally, we found that VEGFR1 was expressed in the majority of cavernous malformations. VEGFR1 is expressed by vascular endothelial and non-endothelial cells. It is induced during vessel growth and remodeling, is required for endothelial cell survival and is upregulated in malignancies (Zhang et al. 2010). VEGFR1 can bind to VEGFA as a negative regulator, serving as a decoy receptor (Weddell et al. 2017). In cavernous malformations, it is possible that VEGFR1 is upregulated in response to increased VEGFR2 and the VEGFA interactions is a method to self-limit growth and rapid expansion. These interactions may be essential for endothelial cell survival even without directly contributing to angiogenesis (Zhang et al. 2010). Previous studies have investigated immunohistochemical staining of VEGFR in orbital vascular tumors (Table2).

The first reports on immunohistochemical staining in orbital cavernous malformations found that 0/9 lesions were positive for VEGFR1 but 9/9 stained for VEGFR2 (Nagasaka et al. 2007). Other studies found that 3/11 (27%) of cavernous malformations expressed VEGF but these studies did not evaluate for VEGFR (Gupta et al. 2012). Rootman et al. performed immunohistochemical staining on 10 cavernous malformations. Of these lesions, 10/10 (100%) demonstrated positive staining with VEGFR1 and 6/10 (60%) demonstrated staining with VEGFR2 (Rootman et al. 2014). These results varied from Nagasaka et al. which found higher rates of VEGFR2 than VEGFR1 expression (Nagasaka et al. 2007).

In this study, the prevalence of VEGFR1 and 2 expression in cavernous malformations was similar to prior studies. Atchison *et al.* was the first to describe VEGFR1 and VEGFR2 expression in lymphatic malformations (Atchison et al. 2016). Their results found higher VEGFR1 and VEGFR2 (6/7 VEGFR1 and 7/7 VEGFR2) compared to our study. This may suggest the possibility of variable VEGFR expression in lymphatic malformations. However, further studies are required to better evaluate the role of VEGFRs and anti-VEGF treatment in lymphatic malformations given the small sample size tested.

Intralesional anti-VEGFA therapy has been used for periocular epithelial hemangiomas as well as intravitreal injections for retinal and choroidal hemangiomas (Mandal et al. 2011, Sagong et al. 2009, Chelala et al. 2013, Kahana et al. 2012). The use of anti-VEGFA for orbital vascular lesions is relatively novel with only limited cases reported. In one case, a cavernous malformation located in the right orbital apex was treated with intralesional bevazicumab (Sweeney et al. 2016). Followed for 2.5 years after the injection, the patient experienced radiographic reduction in lesion size, subjective improvement in dyschromatopsia and complete resolution of visual field defects. Specifically looking at orbital lymphatic malformations, the combination of bevacizumab and sclerosing agents has been investigated. In one case, the combination of intralesional injections was spaced 6 months apart in a patient with a left orbital lymphatic malformation. Over the next year, the patient experienced improvement in proptosis and regression of the lesion (Mustak et al. 2018). In a case series looking at orbital lymphatic malformations with both macrocystic and microsystic features, a combined injection in the macrocystic components and an injection of bevacizumab into microcytic components resulted reduction in lesion size in two patients (Abdelaziz et al. 2019). These results, however, may be confounded by the simultaneous injection of bleomycin. Other cases of orbital lymphatic malformations have been refractory to intralesional bevacizumab injections and required additional therapy (Gooding et al. 2014). Limitations of the study include generalizability, small sample size, and staining focused only on VEGFR1 and VEGFR. While newer classifications have been developed to categorize orbital lymphatic malformations, many of our cases did not have imaging available for review, precluding commenting on more venous, lymphatic or arterial flow or the cyst size. Our study also does not investigate the temporal relationship of VEGFR expression and lesion progression.

In summary, this study utilizes immunohistochemical staining to demonstrate that cavernous malformations and lymphatic malformations may express VEGFR1 and VEGFR2. While additional studies are required to further investigate the role of VEGF in these lesions, this study may provide initial insight into the expression of these receptors and support alternative treatment regimens.

## Figures and Tables

**Figure:**
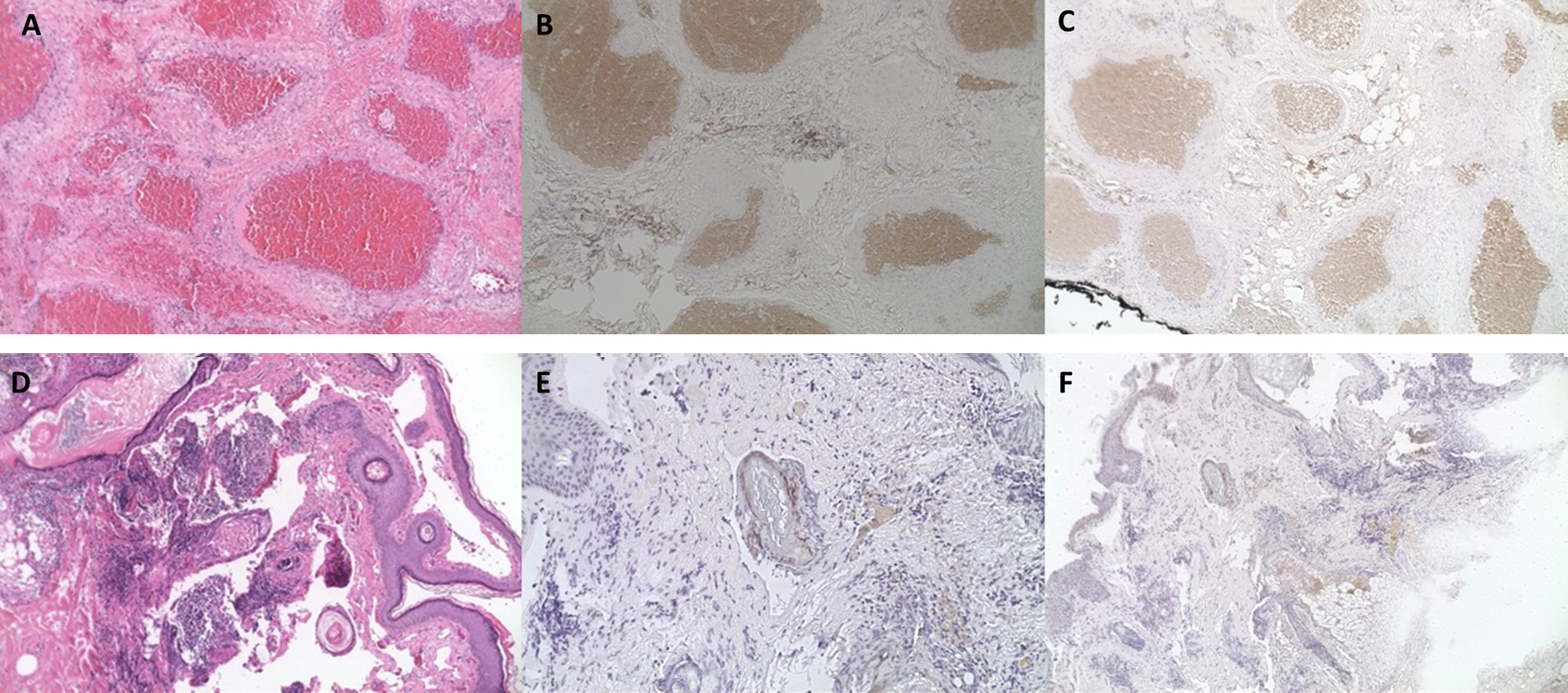
Cavernous malformation hematoxylin and eosin (H&E) immunohistochemical staining at 10× (A)4 with positivity in VEGFR1 (B) and VEGFR2 (C). Lymphatic malformation hematoxylin and eosin (H&E) immunohistochemical staining lymphangioma (D) with positivity in VEGFR1 (E) and VEGFR2 (F).

**Table 1:** Immunohistochemical Staining of Specimens.

Specimen number	Specimen type	VEGFR 1	VEGFR 2
1	Cavernous malformation	+	+
2	Cavernous malformation	+	+
3	Cavernous malformation	+	+
4	Cavernous malformation	−	−
5	Cavernous malformation	+	+
6	Cavernous malformation	+	+
7	Cavernous malformation	+	+
8	Lymphatic malformation	−	+
9	Lymphatic malformation	−	−
10	Lymphatic malformation	+	−

Abbreviations: + = positive, − = negative, VEGFR1: vascular endothelial growth factor receptor 1, VEGFR2: vascular endothelial growth factor receptor 2

**Table 2:** Literature Review of VEGFR1 and VEGFR2 Immunohistochemical Staining of Orbital Vascular Tumors.

Reference	Number of cases	Lesion type	VEGFR1+	VEGFR2+
Nagasaka et al. 2007	n=9	Cavernous malformation	0/9	9/9
Rootman et al. 2014	n=10	Cavernous malformation	10/10	6/10
This study	n=7	Cavernous malformation	6/7	6/7
Atchison et al. 2016	n=7	Lymphatic malformation	6/7	7/7
This study	n=4	Lymphatic malformation	1/3	1/3

Abbreviations: + = positive, VEGFR1: vascular endothelial growth factor receptor 1, VEGFR2: vascular endothelial growth factor receptor 2

## References

[1] Abdelaziz O, Hassan F, Elessawy K, Emad-Eldin S, Essawy R. (2019). Image-Guided Percutaneous Bleomycin and Bevacizumab Sclerotherapy of Orbital Lymphatic Malformations in Children. Cardiovasc Intervent Radiol 42:433–440. 30488306 10.1007/s00270-018-2128-4

[2] Atchison EA, Garrity JA, Castillo F, Engman SJ, Couch SM, Salomao DR. (2016). Expression of Vascular Endothelial Growth Factor Receptors in Benign Vascular Lesions of the Orbit: A Case Series. Ophthalmology 123:209–213. 26481818 10.1016/j.ophtha.2015.09.009

[3] Chelala E, Dirani A, Fadlallah A. (2013). Intravitreal anti-VEGF injection for the treatment of progressive juxtapapillary retinal capillary hemangioma: a case report and mini review of the literature. Clin Ophthalmol Auckl NZ 7:2143–2146. 10.2147/OPTH.S53243 PMC3817061 24204117

[4] Cheng J (2015). Doxycycline sclerotherapy in children with head and neck lymphatic malformations. J Pediatr Surg 50:2143–2146. 26421368 10.1016/j.jpedsurg.2015.08.051

[5] Cursiefen C, Schlötzer-Schrehardt U, Breiteneder-Geleff S, Holbach LM. (2001). Orbital lymphangioma with positive immunohistochemistry of lymphatic endothelial markers (vascular endothelial growth factor receptor 3 and podoplanin). Graefes Arch Clin Exp Ophthalmol Albrecht Von Graefes Arch Klin Exp Ophthalmol 239:628–632. 10.1007/s004170100325 11585321

[6] Elluru RG, Balakrishnan K, Padua HM. (2014). Lymphatic malformations: diagnosis and management. Semin Pediatr Surg 23:178–185. 25241095 10.1053/j.sempedsurg.2014.07.002

[7] Gooding C, Meyer D. (2014). Intralesional bleomycin: a potential treatment for refractory orbital lymphangiomas. Ophthal Plast Reconstr Surg 30: e65–67. 10.1097/IOP.0b013e31829bb4a9 24025999

[8] Gupta A, Prabhakaran VC, Dodd T, Davis G, Selva D. (2012). Orbital cavernous haemangiomas: immunohistochemical study of proliferative capacity, vascular differentiation and hormonal receptor status. Orbit Amst Neth 31:386–389. 10.3109/01676830.2012.711887 23088382

[9] Harris GJ. (1999). Orbital vascular malformations: a consensus statement on terminology and its clinical implications. Am J Ophthalmol 127:453–455. 10218699 10.1016/s0002-9394(99)00048-3

[10] Kahana A, Lee BJ, Flint A, Elner VM. (2012). Periocular Epithelioid Hemangioma: Response to Bevacizumab and Vascular Pathogenesis. Arch Ophthalmol 130:1209–1212. 22965602 10.1001/archophthalmol.2012.572 PMC5018828

[11] Macintosh PW, Yoon MK, Fay A. (2014). Complications of Intralesional Bleomycin in the Treatment of Orbital Lymphatic Malformations. Semin Ophthalmol 29:450–455. 25325873 10.3109/08820538.2014.959617

[12] Mandal S, Naithani P, Venkatesh P, Garg S. (2011). Intravitreal bevacizumab (avastin) for circumscribed choroidal hemangioma. Indian J Ophthalmol 59:248–251. 21586854 10.4103/0301-4738.81051 PMC3120252

[13] Mustak H, Ugradar S, Goldberg R, Rootman D. (2018). Bevacizumab and Bleomycin combination for treatment of orbital lymphatico-venous malformation recalcitrant to sclerosing therapy alone. Clin Experiment Ophthalmol 46:815–816. 29369465 10.1111/ceo.13159

[14] Nagasaka M, Naganuma H, Satoh E. (2007). Growth Potential of Orbital Cavernous Hemangioma Suggested by Vascular Endothelial Growth Factor and its Receptor Flk-1. Neurol Med Chir (Tokyo) 47:5–10. 17245007 10.2176/nmc.47.5

[15] Rootman DB, Heran MKS, Rootman J, White VA, Luemsamran P, Yucel YH. (2014). Cavernous venous malformations of the orbit (so-called cavernous haemangioma): a comprehensive evaluation of their clinical, imaging and histologic nature. Br J Ophthalmol 298:880–888. 10.1136/bjophthalmol-2013-304460 24627253

[16] Sagong M, Lee J, Chang W. (2009). Application of Intravitreal Bevacizumab for Circumscribed Choroidal Hemangioma. Korean J Ophthalmol 23:127–131. 19568366 10.3341/kjo.2009.23.2.127 PMC2694292

[17] Shields JA, Shields CL, Scartozzi R. (2004). Survey of 1264 patients with orbital tumors and simulating lesions: The 2002 Montgomery Lecture, part 1. Ophthalmology 111:997–1008. 15121380 10.1016/j.ophtha.2003.01.002

[18] Shoji MK, Shishido S, Freitag SK. (2020). The Use of Sirolimus for Treatment of Orbital Lymphatic Malformations: A Systematic Review. Ophthal Plast Reconstr Surg 36:215–221. 10.1097/IOP.0000000000001518 31990892

[19] Sweeney AR, Chappell M, Khorsand DA, Jian-Amadi A, Francis CE. (2016). Intralesional Injection of Bevacizumab for the Treatment of an Apical Orbital Cavernous Venous Malformation. J Neuro-Ophthalmol Off J North Am Neuro-Ophthalmol Soc 36:389–392. 10.1097/WNO.0000000000000425 27464980

[20] Tugues S, Koch S, Gualandi L, Li X, Claesson-Welsh L. (2011). Vascular endothelial growth factors and receptors: anti-angiogenic therapy in the treatment of cancer. Mol Aspects Med 32:88–111. 21565214 10.1016/j.mam.2011.04.004

[21] Weddell JC, Chen S, Imoukhuede PI. (2017). VEGFR1 promotes cell migration and proliferation through PLCγ and PI3K pathways. Npj Syst Biol Appl 4:1–11. 29263797 10.1038/s41540-017-0037-9 PMC5736688

[22] Wiegand S, Eivazi B, Bloch LM, Zimmermann AP, Sesterhenn AM, Schulze S, Werner JA, (2013). Lymphatic malformations of the orbit. Clin Exp Otorhinolaryngol 6:30–35. 23526369 10.3342/ceo.2013.6.1.30 PMC3604267

[23] Wiegand S, Eivazi B, Zimmermann AP, Sesterhenn AM, Werner JA (2011): Sclerotherapy of lymphangiomas of the head and neck. Head Neck 33:1649–1655. 20737487 10.1002/hed.21552

[24] Woo YJ, Kim CY, Sgrignoli B, Yoon JS. (2017). Orbital Lymphangioma: Characteristics and Treatment Outcomes of 12 Cases. Korean J Ophthalmol 31:194–201. 28534344 10.3341/kjo.2016.0034 PMC5469922

[25] Zhang Z, Neiva KG, Lingen MW, Nor JE. (2010). VEGF-dependent tumor angiogenesis requires inverse and reciprocal regulation of VEGFR1 and VEGFR2. Cell Death Differ 17:499–512. 19834490 10.1038/cdd.2009.152 PMC2822115

